# Changing accommodation behaviour during multifocal soft contact lens wear using auditory biofeedback training

**DOI:** 10.1038/s41598-020-61904-4

**Published:** 2020-03-19

**Authors:** Sandra Wagner, Frank Schaeffel, David Troilo

**Affiliations:** 1 Institute for Ophthalmic Research, Eberhard Karls University Tuebingen, Elfriede-Aulhorn-Str. 7, 72076 Tuebingen, Germany; 2 Section Neurobiology of the Eye, Institute for Ophthalmic Research, Eberhard Karls University Tuebingen, Elfriede-Aulhorn-Str. 7, 72076 Tuebingen, Germany; 3 State University of New York, College of Optometry, 33 West 42nd Street, New York, NY 10036 United States

**Keywords:** Autonomic nervous system, Visual system, Refractive errors

## Abstract

Biofeedback training has been used to access autonomically-controlled body functions through visual or acoustic signals to manage conditions like anxiety and hyperactivity. Here we examined the use of auditory biofeedback to improve accommodative responses to near visual stimuli in patients wearing single vision (SV) and multifocal soft contact lenses (MFCL). MFCLs are one evidence-based treatment shown to be effective in slowing myopia progression in children. However, previous research found that the positive addition relaxed accommodation at near, possibly reducing the therapeutic benefit. Accommodation accuracy was examined in 18 emmetropes and 19 myopes while wearing SVCLs and MFCLs (centre-distance). Short periods of auditory biofeedback training to improve the response (reduce the lag of accommodation) was performed and accommodation re-assessed while patients wore the SVCLs and MFCLs. Significantly larger accommodative lags were measured with MFCLs compared to SV. Biofeedback training effectively reduced the lag by ≥0.3D in individuals of both groups with SVCL and MFCL wear. The training was more effective in myopes wearing their habitual SVCLs. This study shows that accommodation can be changed with short biofeedback training independent of the refractive state. With this proof-of-concept, we hypothesize that biofeedback training in myopic children wearing MFCLs might improve the treatment effectiveness.

## Introduction

It has been estimated that by 2050, five billion people might be myopic and one billion might suffer from myopia of more than −5 D^1^, significantly increasing associated risks of vision-threatening conditions^2,3^. Among the currently available evidence-based treatments, optical interventions offer effective less invasive means to slow myopia progression^4^. Contact lenses with bi- or multifocal power profiles providing added positive power across the retina or in the periphery have been used successfully to reduce myopia progression rates^5–11^. By reducing hyperopic peripheral defocus and thus moving the peripheral image forward, possibly even creating myopic defocus, the stimulus for eye elongation is thought to be reduced. Visual performance with bi- and multifocal soft contact lenses was previously investigated in pre-presbyopic subjects with respect to the quality of vision^12–14^ and changes in the accommodation behaviour^7,15–22^. A recent investigation by Gong *et al*.^23^ showed that children fitted with centre-distance multifocal soft contact lenses with a positive addition of +2.5 D (*Biofinity*, comfilcon A, Cooper Vision) used the lenses’ addition power during near vision to relax their accommodation. This underaccommodation at near could increase the peripheral hyperopic defocus and thus possibly counteract the contact lenses’ therapeutic effect during near vision tasks^23^.

As part of a larger project to examine the visual changes in patients wearing multifocal soft contact lenses, we aimed to determine (1) how well accommodation accuracy could be trained using auditory biofeedback in emmetropic and myopic young adults wearing their habitual lens correction; (2) whether there are differences in accommodation while wearing single vision or multifocal soft contact lenses; and, (3) whether accommodation biofeedback training can be used to improve accommodation in multifocal soft contact lens wear. If accommodation behaviour with multifocal soft contact lenses can be successfully trained by biofeedback techniques to reduce the use of the positive addition power, the efficacy of these lenses for myopia management might possibly be improved.

Earlier attempts have been made to alter accommodation voluntarily using auditory biofeedback^24^. Based on the hypothesis that myopia development results from an accommodative spasm^25–27^, the aim of these attempts was to reduce accommodation and thus reduce the myopic refractive error^26–30^. Based on current defocus theories of myopia development and management^31,32^, we tested the application of auditory biofeedback training to improve accommodative accuracy and reduce accommodative lag under habitual correction conditions and during multifocal lens wear. We hypothesize that this may increase the efficacy of multifocal lenses and help reduce myopia progression.

## Results

### Subjects characteristics

A total of 37 subjects of which 18 were emmetropic (age 22.11 ± 2.05 years) and 19 myopic (age 21.95 ± 1.72 years), participated in this study. Mean spherical equivalent refractive error of the right eye (±SD) was 0.03 ± 0.30 D in emmetropic and −2.53 ± 0.99 D in myopic participants. Best corrected distance Snellen visual acuity (VA) was at least 6/6 (20/20) in each eye and astigmatic refractive error was ≤0.75 D. The right eye’s accommodation amplitude was (mean ± SD): myopes: 11.89 ± 2.14 D; emmetropes: 10.99 ± 1.80 D.

### Effects of biofeedback training with habitual correction

Accommodation behaviour in emmetropes and myopes in this study was similar at the start of the study. Baseline measurements revealed the lag of accommodation to significantly increase with increasing accommodation demand (F_2,105_ = 16.449, p < 0.001; Table 1) while the refractive state of the participants did not show a significant effect on the accommodative lag (F_1,105_ = 0.381, p = 0.538).

**Table 1:** Accommodation training results in habitual lens wear.

Accommodation demand	Lag of accommodation, D					
	Emmetropic group (n = 18)			Myopic group (n = 19)		
	pre-lag	post-lag	∆lag	pre-lag	post-lag	∆lag
2.5 D	−0.32 ± 0.26	−0.31 ± 0.24	−0.01 ± 0.22	−0.28 ± 0.22	−0.21 ± 0.16	−0.07 ± 0.22
3.0 D	−0.46 ± 0.30	−0.40 ± 0.30	−0.06 ± 0.19	−0.39 ± 0.22	−0.27 ± 0.22	−0.12 ± 0.19
4.0 D	−0.72 ± 0.49	−0.63 ± 0.45	−0.09 ± 0.33	−0.72 ± 0.35	−0.41 ± 0.37	−0.31 ± 0.33

Accommodative lag (mean ± SD) at baseline (pre-lag), after the training (post-lag), and training effects (Δlag = pre-lag − post-lag) in emmetropic and myopic subjects while wearing habitual contact lenses. Negative values for Δlag indicate an improvement of accommodation accuracy after training.

**Figure 1:**
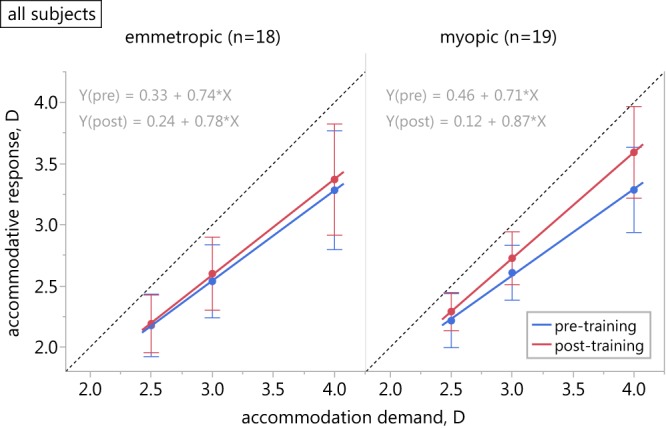
Effect of biofeedback training with habitual correction. Accommodation stimulus-response function of emmetropic (left) and myopic participants in habitual lens wear (right) before (blue) and after the biofeedback training (red). Error bars represent SD of the mean; the dashed line has a slope of 1.

In both refractive groups, the variability of the lag of accommodation between the subjects increased with decreasing target proximity (Fig. 1). Accommodative lag was linearly correlated with the distance, reaching statistical significance in emmetropes (emmetropes: Pearson R = −0.9998, p = 0.014, two-tailed; myopes: Pearson R = −0.9960, p = 0.057, two-tailed).

Following biofeedback training, the amount of accommodative lag was significantly reduced (F_1,105_ = 20.974, p < 0.001). Both the refractive state of the subjects (F_1,105_ = 5.377, p = 0.022) and the accommodation demand (F_2,105_ = 3.538, p = 0.033) significantly influenced the training outcome. Myopic subjects were more successful in reducing their accommodative lag with the largest effect at the closest target distance of 4 D (Fig. 1, Table 1).

The slopes of the stimulus-response functions of pre- and post-training condition were significantly different (F_1,35_ = 10.375, p = 0.003), but refraction did not have a significant effect on the slope changes (F_1,35_ = 3.183, p = 0.083).

The microfluctuations of accommodation during the 200 s-biofeedback session significantly increased with increasing accommodation demand (F_2,105_ = 6.927, p = 0.001), with a significant difference between 2.5 D and 4 D (Bonferroni, p = 0.001). However, no significant effect of the refractive state was found (F_1,105_ = 0.000, p = 0.999). Average fluctuation amplitudes in emmetropes were 0.20 ± 0.08 D, 0.23 ± 0.11 D, and 0.30 ± 0.13 D, while values in myopes were 0.22 ± 0.07 D, 0.23 ± 0.05 D, 0.26 ± 0.07 D at 2.5, 3, and 4 D distance, respectively.

In the emmetropic group, seven subjects were able to reduce their lag of accommodation by ≥0.3 D (39% of the group), while in the myopic group, this was achieved by 12 subjects (63%). The values of ∆lag of accommodation in the emmetropic sub-group were −0.13 ± 0.16 D, −0.23 ± 0.17 D, −0.36 ± 0.23 D, and in the myopic sub-group −0.16 ± 0.21 D, −0.15 ± 0.19 D, and −0.48 ± 0.29 D at 2.5, 3, and 4 D target distance, respectively. In responsive myopes, the slope of the stimulus-response function changed from 0.67 ± 0.18 to 0.90 ± 0.23 while the change in the emmetropic sub-group was from 0.68 ± 0.10 to 0.83 ± 0.18.

### Accommodation response in single vision vs. multifocal contact lens wear

The average lag of accommodation during *Biofinity* single vision and *Biofinity* multifocal soft contact lens wear in the two refractive groups at the tested distances is shown in Table 2.

**Table 2:** Average values of lag of accommodation with different contact lens types.

Accommodation demand	Lag of accommodation, D			
	Emmetropic group (n = 18)		Myopic group (n = 19)	
	Single Vision	Multifocal	Single Vision	Multifocal
2.5 D	−0.37 ± 0.25	−1.06 ± 0.38	−0.27 ± 0.34	−1.16 ± 0.38
3.0 D	−0.50 ± 0.28	−1.26 ± 0.39	−0.44 ± 0.35	−1.43 ± 0.35
4.0 D	−0.79 ± 0.34	−1.65 ± 0.46	−0.72 ± 0.44	−1.77 ± 0.37

Mean and standard deviation of the lag of accommodation with *Biofinity* single vision vs. multifocal soft contact lenses at the three tested distances in both study groups.

The amount of the lag of accommodation significantly differed depending on target distance (F_2,210_ = 37.743, p < 0.001) and lens type (F_1,210_ = 314.696, p < 0.001). No significant influence of the refractive state of the subjects was given (F_1,210_ = 0.333, p = 0.564), however, a significant interaction effect of refraction and lens type was revealed (F_1,210_ = 4.161, p = 0.043).

The closer the fixation target, the larger the lag of accommodation became in both refractive groups and with both lens types. While the myopic participants showed a lower lag of accommodation in single vision contact lens wear, their lag was larger with multifocal lenses than in their emmetropic counterparts at all tested distances, respectively.

**Figure 2:**
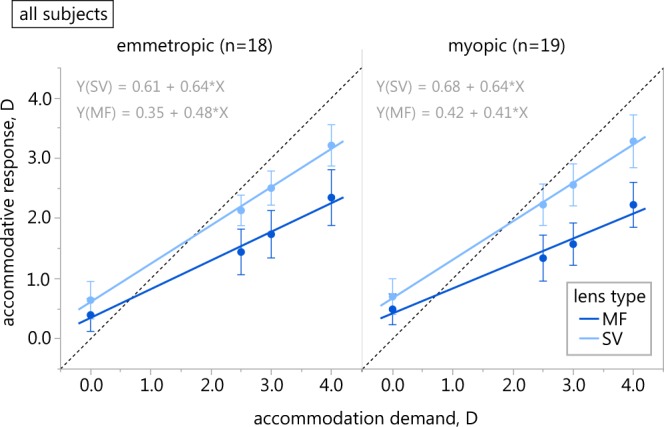
Stimulus-response function in different contact lens wear. Accommodation stimulus-response function of emmetropic (left) and myopic group (right) with single vision (light blue) and multifocal soft contact lenses (dark blue). Error bars denote SD of the mean; the dashed line has a slope of 1.

The average slope of the accommodation stimulus-response functions of both study groups for single vision vs. multifocal lenses, respectively, was 0.64 ± 0.10 vs. 0.48 ± 0.11 in emmetropes and 0.64 ± 0.09 vs. 0.41 ± 0.08 in myopes (Fig. 2).

### Effects of biofeedback training in multifocal contact lens wear

**Figure 3:**
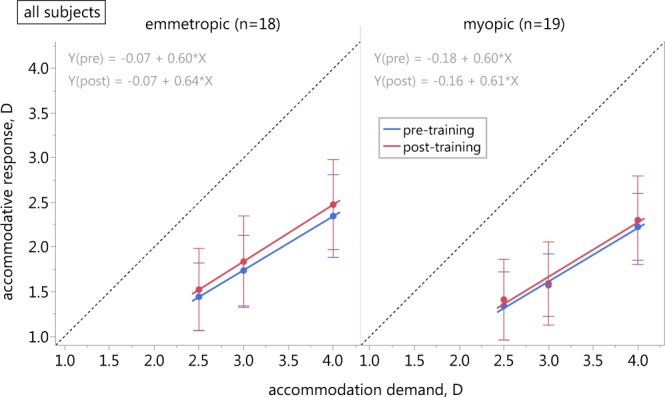
Training effect in multifocal contact lens wear. Accommodation stimulus-response functions of both study groups (emmetropes left, myopes right) with multifocal contact lenses. Error bars denote SD of the mean; the dashed line has a slope of 1.

Baseline accommodative lag with multifocal lenses was not influenced by the subjects’ refractive state (F_1,105_ = 3.013, p = 0.086), but target distance was a factor (F_2,105_ = 22.422, p < 0.001) with larger lags for closer targets. The analysis of the ∆lag of accommodation (Table 3) revealed a statistically, however not clinically significant difference between the lag before vs. after the auditory biofeedback training (F_1,105_ = 7.228, p = 0.008). Neither the distance (F_2,105_ = 0.175, p = 0.840), nor the refractive state (F_1,105_ = 0.676, p = 0.413) had a significant effect on the change of the accommodation response (Fig. 3).

**Table 3:** Training effects in multifocal lens wear.

Accommodation demand	Lag of accommodation, D					
	Emmetropic group (n = 18)			Myopic group (n = 19)		
	pre-lag	post-lag	∆lag	pre-lag	post-lag	∆lag
2.5 D	−1.06 ± 0.38	−0.98 ± 0.46	−0.08 ± 0.27	−1.16 ± 0.38	−1.09 ± 0.45	−0.07 ± 0.26
3.0 D	−1.26 ± 0.39	−1.16 ± 0.51	−0.10 ± 0.39	−1.43 ± 0.35	−1.41 ± 0.47	−0.02 ± 0.24
4.0 D	−1.65 ± 0.46	−1.52 ± 0.50	−0.13 ± 0.33	−1.77 ± 0.37	−1.70 ± 0.50	−0.07 ± 0.34

Lag of accommodation before (pre-lag) and after the biofeedback training (post-lag) and the training effect (Δlag = pre-lag − post-lag) in the two study groups while wearing multifocal contact lenses (mean ± SD). Negative values for Δlag indicate an improvement of accommodation accuracy after training.

The microfluctuations of the biofeedback measurements did not depend on the accommodative demand (F_2,105_ = 1.979, p = 0.143), however there was significantly less microfluctuation in the myopic subjects (F_1,105_ = 4.690, p = 0.033). At 2.5 D and 3 D, the microfluctuations of emmetropes and myopes only differed marginally (2.5 D: 0.27 ± 0.09 D vs. 0.23 ± 0.07 D; 3 D: 0.26 ± 0.10 D vs. 0.25 ± 0.09 D for emmetropes and myopes, respectively), while at 4 D, the emmetropes’ microfluctuations exceeded those of the myopes with 0.32 ± 0.11 D vs. 0.25 ± 0.08 D.

Eight of the emmetropic (44%) and eight of the myopic subjects (42%) were responsive to the biofeedback training, i.e. showed a reduction of the accommodative lag by 0.3 D or more. These 16 subjects were asked to return for re-evaluation five to eight days after the training; twelve (five emmetropes, seven myopes) returned. Supplementary Fig. S1 illustrates the individual stimulus-response functions of the responsive subjects who participated in the control measurements one week after training. While most subjects showed continuous reductions in their accommodative lag over the three measurement times in at least one distance, a few did not reproduce the training effects after one week.

The sub-groups’ mean and standard deviation of the lag of accommodation at the different times of measurement are provided in Table 4.

**Table 4:** Lag of accommodation in subjects successfully trained in multifocal contact lens wear.

	Lag of accommodation, D					
	Emmetropic sub-group (n = 5)			Myopic sub-group (n = 7)		
	2.5 D	3.0 D	4.0 D	2.5 D	3.0 D	4.0 D
pre-training	−1.32 ± 0.47	−1.57 ± 0.46	−1.86 ± 0.25	−1.11 ± 0.33	−1.37 ± 0.33	−1.82 ± 0.43
post-training	−0.95 ± 0.73	−1.21 ± 0.61	−1.45 ± 0.49	−0.78 ± 0.31	−1.11 ± 0.36	−1.49 ± 0.56
control measurement	−0.71 ± 0.88	−0.88 ± 0.79	−1.21 ± 0.66	−0.72 ± 0.38	−1.09 ± 0.73	−1.27 ± 0.77

Values of the lag of accommodation (mean ± SD) in the five emmetropic and seven myopic responsive subjects (i) in the baseline reading, (ii) in the post-training measurement without biofeedback, and (iii) in the control measurement, also without biofeedback, recorded five to eight days after the training in multifocal contact lens wear.

**Figure 4:**
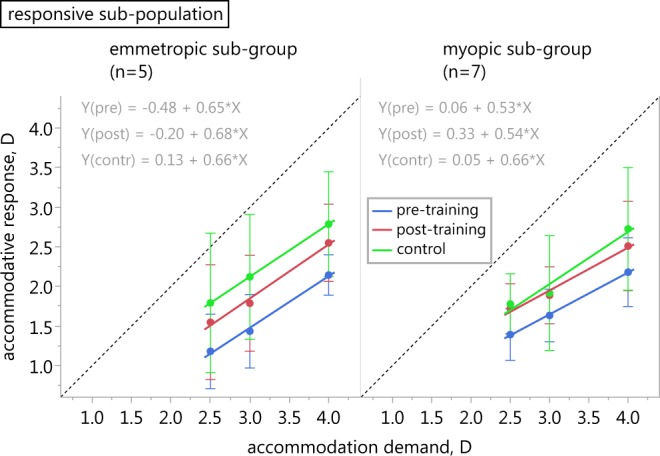
Accommodation in responsive subjects with control measurement. Stimulus-response functions of responsive emmetropic (n = 5, left) and myopic subjects (n = 7, right) at baseline (blue), after the biofeedback training (red), and in the control measurement one week later (green) in multifocal contact lens wear, respectively. The dashed line has a slope of 1; error bars are SD of the mean.

Pre-training values of the lag of accommodation were larger in the emmetropic participants of the sub-group at all distances. However, emmetropes still exhibited a larger reduction of the lag in consequence of the training (∆lag (=pre-lag − post-lag) emmetropes: −0.37 ± 0.34 D, −0.35 ± 0.24 D, −0.41 ± 0.41 D; myopes: −0.33 ± 0.20 D, −0.26 ± 0.19 D, −0.33 ± 0.22 D, at 2.5, 3, and 4 D, respectively). In the control measurement, the emmetropes’ performance was also better than in myopes as they achieved a larger reduction of their lag compared to both the baseline reading and the post-training reading. Nevertheless, in both refractive sub-groups, the lag of accommodation was on average further reduced in the control measurement after about one week compared to the two previous readings (Fig. 4).

## Discussion

In this paper we examine the accommodative response while subjects wore either single vision distance correction or multifocal contact lenses for the management of myopia progression. We aimed to assess the ability to alter accommodation voluntarily to evaluate the use of auditory biofeedback training to improve the myopia control treatment effect of multifocal contact lenses in progressing myopic children.

In contrast to previous investigations, in this study we did not find a significant difference between the baseline accommodative lag of emmetropic and myopic subjects wearing their habitual soft contact lens correction^16,33–35^. A possible reason could be the lower range of myopic refractive error in our subjects.

We did find a significant increase in the lag of accommodation in multifocal compared to single vision lens wear in both refractive groups, indicating that the subjects use the positive addition power to relax their accommodation during near vision, confirming several previous studies^15,21,23^. However, while average lags of accommodation of 1.12 D and 0.82 D with a +1.5 D and a +3 D add power multifocal lens, respectively, were measured in a group of 24 young adult myopes at 3 D distance^15^, even larger lags (1.26 D in emmetropes and 1.43 D in myopes) were measured in the present study at the same demand with an add power of +2.5 D. The average refractive state of 15 myopic children wearing a +2.5 D multifocal lens was found to be 1.75 D for an accommodation demand of 4 D^23^. The lags at 4 D in the current trial are in a slightly lower range with lags of accommodation of 1.65 D in emmetropic and 1.77 D in myopic participants. Deviations of our results from those reported by Kang and Wildsoet could be explained by the usage of different lens types and addition powers, as they have an impact on the lenses’ power profile^36,37^, as well as by applying different measurement devices and conditions (binocular vs. monocular viewing). A reduced accommodation response with multifocal contact lenses causes concern regarding the lenses’ efficiency in myopia control: Relaxation of accommodation during near vision would result in more lag (hyperopic defocus) on axis, and possibly even more on the peripheral retina – a condition that might trigger greater axial elongation and myopia progression^38^.

In contrast, however, other studies did not find significant differences in the accommodation responses between single vision and multifocal contact lenses^17–19^, and in one study a lead of accommodation was reported for bifocal contact lens wear^16^. The effect of multifocal lenses on the accommodation response was analysed using lenses with centre-near design in some of these studies^17–19^, which might have affected accommodative behaviour and requires further examination. The lead of accommodation reported by Tarrant *et al*.^16^ can be explained by the unconventional definition of the error of accommodation used in that study.

In our study of biofeedback training with habitual lens wear, we found that the myopes exhibited a larger reduction of accommodative lag compared to emmetropes, even though the two groups did not differ at baseline in accommodative lag. In contrast to this initial biofeedback training, in which 63% of the myopic subjects were responsive compared to 39% of the emmetropes, only 42% of the myopes and 44% of the emmetropes reduced their lag of accommodation by ≥0.3 D in the second training when multifocal lenses were worn. At this time, it is unclear why there should be a difference between refractive groups, as well as why there should be a difference between single vision and multifocal lens conditions. The increased accommodation after training in responsive subjects cannot be explained by an increase of convergence as the changes of the convergence angle between baseline and post-training readings were minimal. Furthermore, pupil sizes were continuously recorded in all cases and carefully analysed. They were always above 5 mm, which was larger than the multifocal zones of the contact lenses^36^, excluding the possibility that vignetting could have had an effect. The pupil sizes increased during biofeedback training, assumingly due to arousal, but differences in pupil diameter between the different stages of the experiment were not significant (e.g. left eye’s pupil size changes (mean ± SD) for training with MFCLs in responsive subjects: ∆_pre-post_ −0.03 ± 0.32 mm, ∆_pre-biofeedback_ −0.18 ± 0.32 mm, ∆_biofeedback-post_ 0.15 ± 0.22 mm, respective values in non-responders: 0.08 ± 0.41 mm, −0.21 ± 0.31 mm, and 0.30 ± 0.31 mm).

Our periods of training were short, and apparently not all subjects respond the same way (see Supplementary Fig. S1). This variability in accommodation and the response to training may be important in the response to the multifocal lens treatment. Longer, repeated periods of training with longer breaks to the post-training reading need to be tested. Altogether, however, four emmetropic and five myopic subjects achieved a reduction of their lag of accommodation in both trainings, with habitual correction and with multifocal contact lenses indicating that even short periods of biofeedback training can effectively alter accommodative performance through multifocal treatment lenses.

Further analysis of subjects responding to short periods of accommodative biofeedback training re-measured one week after training shows that the emmetropes, although exhibiting larger lags of accommodation than myopes at baseline, achieved a greater reduction of their lags. The emmetropic sub-group’s training success is even more striking as their average baseline lag is larger at all dioptric distances (Table 4) compared to the average baseline values of the entire emmetropic group wearing multifocal contact lenses (Table 3). Nevertheless, the average performance of the twelve tested responsive subjects, both emmetropic and myopic, reveals a continuous reduction of the lag of accommodation from pre-training to control measurement after one week. We can therefore assume that most of these subjects were able to adjust their accommodative response following training. This could reflect better accommodative control and adaptation, which was still present after about one week and without further training. Accommodation training through multifocal lenses, however, might require longer training periods, particularly in myopes, than provided in this study as adaptation to multifocals was reported to not change after two weeks^15^ and was even hypothesized to be unlikely in pre-presbyopic subjects in the long-term^22^.

Regarding the microfluctuations of accommodation during the biofeedback training with habitual correction (myopes) or no correction (emmetropes) on the one hand and multifocal lenses (both groups) on the other, a remarkable finding was that with habitual correction, microfluctuations were influenced by the target distance, but not by the subjects’ refractive state. The opposite was observed with the multifocal lenses. We speculate that the increased microfluctuations of emmetropes during biofeedback with multifocal lenses at 4 D could be related to their better performance. The decreased training effects in the myopic group with multifocal lenses could result from the conflicting signals induced by the different lens zones. There is evidence that stimulus to accommodate is not restricted to the fovea but extends into the peri-fovea^39,40^ which could explain the inter-individual variability in training outcome. Prolonged training durations and multiple training sessions might be necessary to transfer the myopes’ responsivity in single vision to multifocal lens wear.

This study demonstrates that accommodation is an important consideration in optical treatments for myopia management and requires further study. While this study provides proof-of-concept that accommodative behaviour can be modified possibly to improve the therapeutic effect of multifocal contact lenses, a number of important questions about the duration and frequency of the training necessary in young myopes, as well as the efficacy of training in a paediatric population need to be addressed. Possible limitations in this study are that photorefraction does not provide a clear distinction between spurious artefacts resulting from ocular spherical aberration changes and genuine accommodative errors^41^, the limited number of accommodation demands tested and used for assessing stimulus-response functions, as well as the study population’s age, which does not represent the biofeedback training’s actual target group.

## Conclusion

The current investigation assessed the efficacy of auditory biofeedback training to improve the accommodation response through multifocal soft contact lenses with the aim to prevent relaxation of accommodation. We found that both emmetropes and myopes tend to use the add power of multifocal lenses to reduce the accommodative response to near point demands, which could be adjusted in some individuals by a brief period of biofeedback training. Differences between myopes and emmetropes in their accommodative behaviour and plasticity in response to training were observed while wearing multifocal lenses. Whether this variability reflects differences in efficacy of the lenses for reducing myopia progression is unknown and remains to be determined. We find that even a short period of accommodative biofeedback training can reduce the lag of accommodation by 0.3 D or more in 44% of emmetropic and 42% of myopic young adults tested, with some subjects showing a prolonged training effect over a period of one week. This may be improved by longer periods of repeated training and needs to be determined. We are currently testing whether the application of biofeedback training in myopic children treated with multifocal contact lenses (and possibly orthokeratology as well) for the reduction of myopia progression can improve treatment efficacy by increasing the effect of the positive addition lens during near vision.

## Methods

### Subjects

Volunteers aged between 18 to 25 years were recruited from the student body of the University of Tuebingen, Germany. They were informed about purpose, procedure, and possible risks of the trial and subsequently gave their written informed consent. The study followed the tenets of the declaration of Helsinki and was approved by the Institutional Review Board of the medical faculty of the University of Tuebingen.

### Procedure of measurements

**Figure 5:**
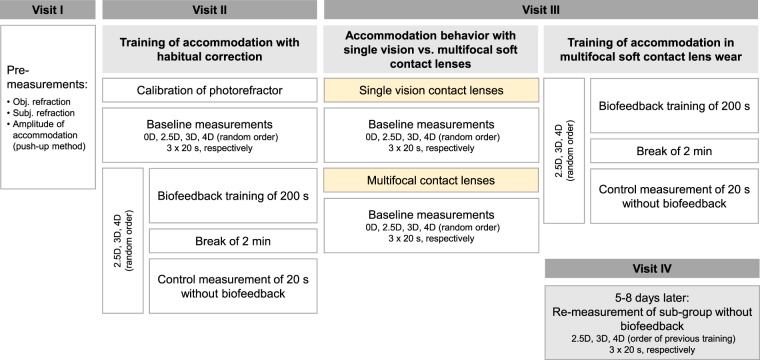
Description of trial procedure. Overview of first, second, third, and forth visit with description of respective measurement procedure.

During a first visit (Fig. 5), the anamnesis including questions on medical and vision history, and baseline measurements of objective and subjective refraction (using autorefractor and digital phoropter), and the amplitude of accommodation (using the push-up method) were measured in all subjects. Monocular and binocular best corrected VA was determined in the subjective refraction following the rule of least negative correction for maximum achievable VA. Subjects with spherical equivalents in the range of ±0.5 D were assigned to the emmetropic group. In myopic subjects, best corrected VA was determined while the subjects wore their own single vision soft contact lens correction. In case of a monocular Snellen VA below 6/7.5 (20/25), they were fitted with appropriate spherical daily disposable contact lenses with the spherical equivalent (*1-Day Acuvue Moist*, etafilcon A, Johnson & Johnson) for the first measurement session. Accommodation response was measured in the vertical meridian using an eccentric infrared (IR) photorefractor with a sampling rate of 80 Hz. The photorefractor was calibrated individually during the second visit using trial lenses^42–44^, as also recommended recently^45^.

The main measurements were performed on separate visits and were divided into three parts (Fig. 5): (1) Auditory biofeedback training to reduce accommodative lag was assessed in the two refractive groups, while myopes wore their habitual soft contact lens correction or spherical daily disposables (visit 2). (2) Baseline accommodation stimulus-response functions were determined in both refractive groups while myopic subjects wore single vision distance correction and emmetropes wore plano lenses, then repeated while they wore multifocal soft contact lenses (centre-distance with +2.5 D adds). (3) Feedback training of accommodation while wearing the multifocal contact lenses was assessed (both (2) and (3) during visit 3) and accommodative measurements with multifocals were repeated in responsive subjects five to eight days later (visit 4).

### Biofeedback training in single vision contact lens wear

Room illuminance was adjusted to about 120 lux to obtain pupil sizes of 4 mm or more for accurate recordings with the eccentric IR photorefractor. Myopic subjects used their own single vision or daily disposable single vision soft contact lenses, while emmetropes were not given any contact lenses. Near fixation targets were shown on a high resolution QXGA TFT LCD display (Adafruit Qualia 9.7′′ DisplayPort Monitor, Adafruit, New York City, USA, luminance 2 cd/m², resolution 2048 × 1536 pixels), positioned in the midline between both eyes. The display was moved on an optic track to provide different accommodation demands. The far target was presented on a second monitor at 4.35 m distance. Words of five letter length in Sloan Font^46^ and scaled in size, corresponding to a VA demand of about Snellen 6/9.5 (20/32) for each target distance, were presented in white font on a black background, with a central coloured letter. They changed with a frequency of 2 Hz according to the rapid serial visual presentation paradigm and kept the same position on the display. To create monocular viewing conditions, the left eye was covered by an IR transmitting filter (RG695, SCHOTT AG, Mainz, Germany) allowing binocular accommodation readings. Subjects were asked to fixate the coloured letter of the changing words and to keep it as sharply as possible at all times. Three 20 s-baseline readings of the accommodation response were taken at four target distances with 0, 2.5, 3, and 4 D accommodation demands, consecutively chosen in random order. Subsequently, the biofeedback training was performed (Fig. 5). The biofeedback tone was produced using custom-made software driven by the output of the photorefractor as described previously^43^. The real-time accommodation response was translated into an auditory signal, continuously using the mean of 25 readings of the 80 Hz recordings. Higher frequencies corresponded to higher accommodation response values. Subjects were alternately provided with their auditory biofeedback tone and a target tone that matched the respective tested dioptric distance, either 2.5, 3, or 4 D. The subject’s task was to adapt the accommodation response to match the pitches of biofeedback and target tone during a period of 200 s. After a break of two minutes to relax their eyes, subjects were asked to reproduce the accommodation response without being provided with biofeedback for a measurement period of 20 s. Subjects were specifically instructed to refrain from squinting and cross fusing. The same training protocol (200 s-biofeedback training − 2 min-break − 20 s-recording without biofeedback) was followed at each near target distance, chosen in random order. Prior to the training, subjects were given a familiarization phase with the biofeedback procedure. For data analysis, the median of the three baseline recordings was calculated for each distance and the effect of the biofeedback training was assessed by comparing the accommodative lag (demand-response) before and after training (∆lag of accommodation = pre-lag − post-lag) and the slope changes of the stimulus-response functions (∆slope = pre-slope − post-slope). Only accommodation responses of the right eyes were analysed.

### Measurements of accommodation response in *Biofinity* single vision vs. multifocal contact lens wear

Accommodation responses were measured while subjects were first wearing single vision and secondly multifocal soft contact lenses with centre-distance design. Single vision lenses (*Biofinity*, comfilcon A, Cooper Vision) with appropriate distance correction were chosen for myopic participants, while emmetropes received plano lenses. For measurements in multifocal lens wear, *Biofinity* multifocal contact lenses (addition +2.5 D) with distance correction in myopes and plano power in emmetropes were fitted on the left eye, while *Biofinity* single vision lenses with distance correction in myopic, and plano power in emmetropic subjects were fitted on the right eye. The IR transmitting filter was placed in front of the right eye to allow photorefractor recordings. Subjects were asked to consecutively fixate targets at 0, 2.5, 3, and 4 D dioptric distance in random order. With both lens designs, three measurements of 20 s each were taken at each distance and the median was determined, respectively. Fixation targets and presentation paradigm were the same as in the prior measurement part.

### Biofeedback training in multifocal contact lens wear

After these baseline readings, each subject performed the biofeedback training while wearing the multifocal contact lens in the left eye and the single vision lens in the right eye, covered by the IR transmitting filter. The study protocol of Part 1 was followed, consisting of 200 s-biofeedback training, a 2-min break, and a 20 s-control measurement without biofeedback. The training was performed in random order at 2.5, 3, and 4 D distance.

### Evaluation of responsivity of subjects

For both biofeedback trainings, in habitual and multifocal lens wear, subjects were categorized as responsive in case of a reduction of the accommodation lag by ≥0.3 D^43^. In subjects responsive to the training with multifocal lenses, a re-measurement of accommodation after about one week was performed while wearing the multifocal contact lens on the left, and the single vision lens with the filter on the right eye. Three 20 s-recordings without biofeedback were taken at the three distances, chosen in the order of the previous training, and the median was calculated for the following analysis, respectively.

### Data processing

Accommodation measurements were filtered for blink artefacts using a script written in Matlab (The Mathworks GmbH, Ismaning, Germany). Microfluctuations of accommodation during the 200 s-biofeedback training were quantified using the responses’ standard deviation^47^.

### Statistical analysis

Statistical analysis was performed using IBM SPSS Statistics 24 (IBM Deutschland GmbH, Ehningen, Germany) and JMP 14 (SAS Institute GmbH, Heidelberg, Germany). Data was approximately normally distributed (Kolmogorov-Smirnov test). Univariate ANOVA with the factors refraction (emmetropic, myopic) and target distance (2.5, 3, 4 D) for analysis of baseline lag, training effects (∆lag), and microfluctuations, and with the additional factor lens type for the evaluation of accommodation with single vision vs. multifocal contact lenses was performed. Slope changes of the stimulus-response function (∆slope) were assessed in a univariate ANOVA with the factor refraction.

## Supplementary information

Supplementary Figure S1.

## Data Availability

The datasets generated during and/or analysed during the current study are available from the corresponding author on reasonable request.
