# Supplementary material for: Changing accommodation behaviour during multifocal soft contact lens wear using auditory biofeedback training
Source: Sci Rep. 2020 Mar 19;10:5018. doi: 10.1038/s41598-020-61904-4 (PMC7081315; doi:10.1038/s41598-020-61904-4)
Supplement: Supplementary file 1 — Supplementary Figure S1. [file 41598_2020_61904_MOESM1_ESM.pdf]

Changing accommodation behaviour during multifocal soft contact lens wear using auditory biofeedback training

Sandra Wagner*, Frank Schaeffel, David Troilo

Supplementary Information

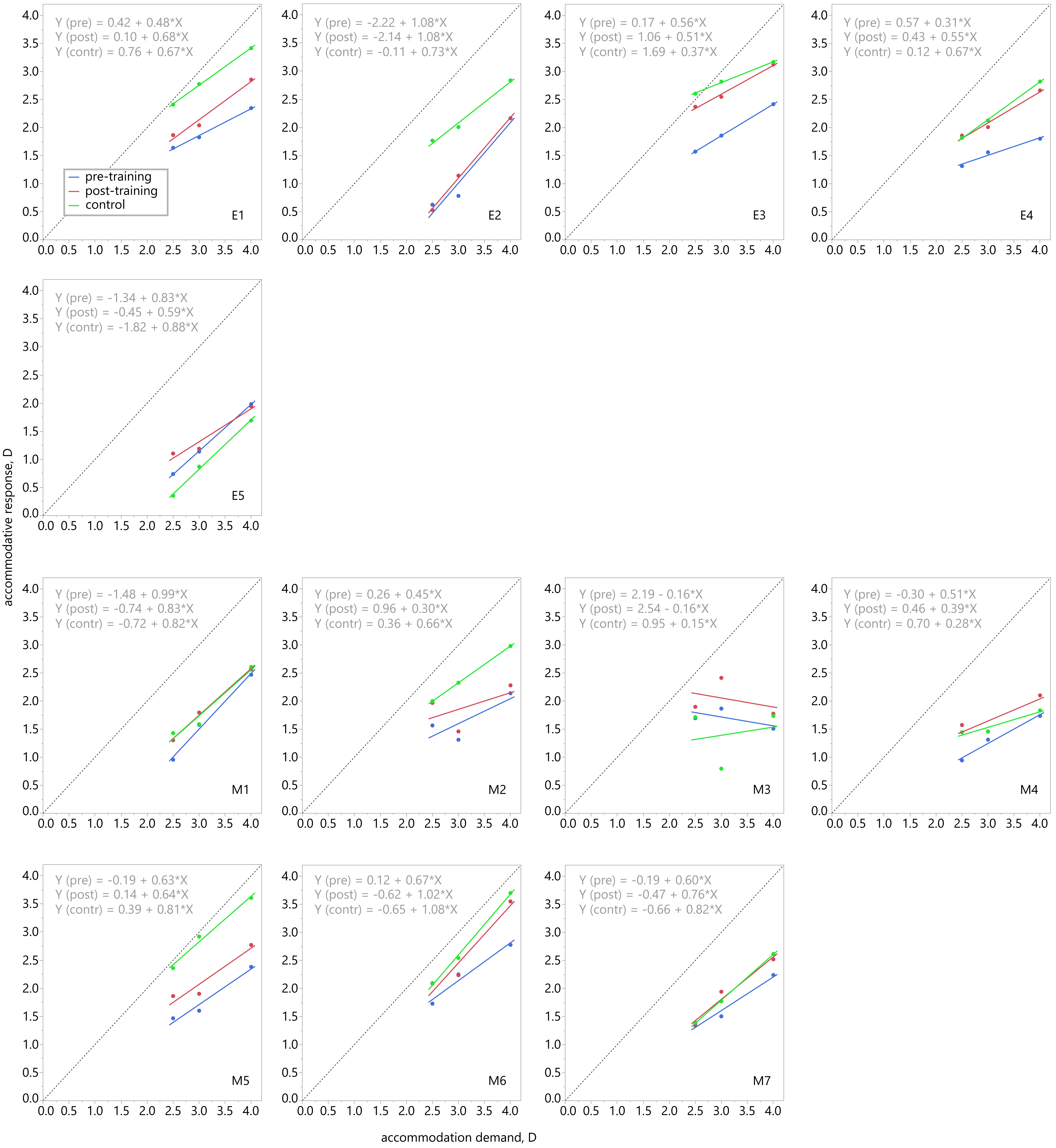

Supplementary Fig. S1: Individual stimulus-response functions of responsive sub-group in multifocal contact lens wear. Individual accommodation stimulus-response function in emmetropic (E1 to E5, top) and myopic (M1 to M7, bottom) subjects of the responsive sub-group at baseline (blue), after the biofeedback training (red), and in the control measurement five to eight days after training (green) in multifocal contact lens wear, respectively. The dashed line has a slope of 1.
